# Radiographers’ self-perceived knowledge regarding infection control in the Free State province during the COVID-19 pandemic

**DOI:** 10.4102/hsag.v31i0.3146

**Published:** 2026-01-14

**Authors:** Ida-Keshia Sebelego, Je’nine Horn-Lodewyk

**Affiliations:** 1 Department of Clinical Sciences, Faculty of Health and Environmental Sciences, Central University of Technology, Bloemfontein, South Africa; 2 Pacific Radiology, Dunedin, New Zealand

**Keywords:** knowledge, training, infection control, qualified radiographers, diagnostic radiography students

## Abstract

**Background:**

Radiographers are regarded as essential healthcare workers. Therefore, knowledge and training regarding infection control are vital to minimise the potential risk of infections to patients.

**Aim:**

The aim of the study was to investigate the self-perceived knowledge of infection control among qualified radiographers and diagnostic radiography students.

**Setting:**

The study was conducted in three public sector radiology departments during the coronavirus disease 2019 (COVID-19) pandemic.

**Methods:**

A quantitative questionnaire was completed by 106 participants comprising radiographers and students of diagnostic radiography. Data were analysed using SAS version 9.4. The Wilcoxon two-sample and Kruskal-Wallis tests were performed to calculate the difference between the median values and to determine the significant differences between the experience, training cycle of infection knowledge, and knowledge of the participants.

**Results:**

Only 30.2% (*n* = 32) of the participants knew that hands are the most basic mode of transmitting infections, while 98.1% (*n* = 104) knew that long fingernails harbour microbes. The participants’ knowledge of infection control was low to adequate. They rated themselves as having adequate to excellent knowledge about infection control. However, their median score was 60% which demonstrates an adequate level of knowledge. No significant relationship was noted between training and knowledge nor between the years of experience and knowledge.

**Conclusion:**

Considering the findings, radiographers’ and student radiographers’ knowledge of infection control requires improvement. An infection control audit accompanied by in-service training on infection control might encourage radiographers to comply with infection control guidelines and enhance their knowledge.

**Contribution:**

While infection control has been widely studied, fewer studies focus specifically on diagnostic radiography students and radiographers, despite their direct patient contact and exposure to infection risks. The study provides much-needed data for this under-researched group.

## Introduction

South African healthcare professionals are expected to uphold the rights of patients. The Patient Rights Charter states that patients have the right to a safe and healthy environment, which includes protection from infections while in the care of healthcare workers (Health Professions Council of South Africa [HPCSA] 2023). Having the necessary knowledge of infection control, such as standard precautions, is essential for radiographers to apply during medical imaging procedures. Knowledge of infection control directly affects attitudes, practices and adherence to preventive behaviour (Albaqawi et al. 2020; Lee, Kang & You 2021).

Several studies have reported on the knowledge of qualified radiographers in different areas of infection control. Abu Awwad et al. (2023) noted that limited research has been conducted on the knowledge, attitudes and practices (KAP) of computed tomography (CT) radiographers regarding infection control in the CT working environment. Based on their study, the CT radiographers’ knowledge score was 95%, with training contributing to knowledge (Abu Awwad et al. 2023). In another Saudi Arabian study, the radiographers demonstrated a moderate level of knowledge regarding standard precautions, which was considered reasonable. However, their findings emphasised the need for further education to improve radiographers’ knowledge (Gareeballah et al. 2023). Abdelrahman et al. (2017) investigated radiographers’ knowledge of nosocomial infections, standard precautions and infection control practices. They concluded that the radiographers had a moderate level (66.2%) of knowledge, indicating that their knowledge was considered suboptimal and required improvement. Gareeballah et al. (2023) and Abdelrahman et al. (2017) suggested that radiographers should receive training to enhance their knowledge of infection control measures. Furthermore, Nyirenda et al. (2018) also concluded that training would assist radiographers in enhancing their knowledge of infection control after their results revealed that radiographers in Malawi showed a moderate level of knowledge relating to infection control.

During the coronavirus disease 2019 (COVID-19) pandemic, research was conducted on qualified radiographers and radiology healthcare workers, including nurses and support staff, to gather knowledge on COVID-19 infection control (Aljondi et al. 2021; Ezema et al. 2023; Ooi et al. 2023). Zakaria and Mohamed (2023) assessed the knowledge of infection control among radiography students. Overall, the students demonstrated a good understanding of infection control as it was part of the qualification curriculum and specifically discussed in patient care and clinical practice modules. No research could be found that evaluated the knowledge of infection control among both students and qualified radiographers during a pandemic.

This study aimed to determine the knowledge of infection control among qualified and student radiographers during a pandemic, whether they received training, and if such training enhanced their knowledge. The study also aimed to determine whether the position and experience of the qualified and student radiographers positively impacted their knowledge of infection control.

The objectives were to: (1) describe the profile of the participants; (2) determine their knowledge of basic infection control; (3) determine their training history and (4) determine whether their position, experience and training influenced their infection control knowledge.

## Research methods and design

### Study design

A positivist paradigm was employed in this study to understand the knowledge of qualified radiographers and diagnostic student radiographers (Mackenzie & Knipe, 2006). The study employed a descriptive approach and utilised a quantitative methodology. A quantitative questionnaire was compiled and captured in the TurningPoint programme, also known as the clicker system (Turning Technologies, LLC, Youngstown, OH, United States). A clicker system was used successfully previously to evaluate radiographers’ knowledge of a particular topic (Sebelego 2019). Using the clicker system during the research eased the process of data collection and data analysis, as stated by Sebelego (2019).

A questionnaire comprising two sections was compiled based on information from the literature, such as Ehrlich and Coakes (2017), Hilt et al. (2019), Oh (2019), Alharbi et al. (2016), the Centers for Disease Control and Prevention, Ilyas, Burbridge and Babyn (2019), Cimon and Featherstone (2017), Suen et al. (2019), Whitley (2020) and the World Health Organization (WHO 2021) and referred to as the clicker questionnaire. Section A included demographic information, and the questions in Section B were based on a literature review to determine the radiographers’ background knowledge regarding infection control. The clicker questionnaire consisted of 20 questions that explored radiographers’ knowledge and two questions about infection control training. Four questions were based on the participants’ demographic information, including their position in the radiology department and their years of experience in the radiography profession.

### Setting

The study was conducted at three public sector radiology departments in the Free State province in South Africa during the COVID-19 pandemic.

### Study population and sampling technique

The population for this study consisted of qualified radiographers and undergraduate diagnostic radiography students who rotated between general X-rays, fluoroscopy, magnetic resonance imaging (MRI) and CT. A convenience sampling technique was employed, with a sample size of 106 diagnostic radiographers comprising qualified radiographers, community service radiographers, and supplementary and student radiographers (2nd-, 3rd- and 4th-year Bachelor of Radiography in Diagnostics students). First-year radiography students were excluded from the sample as they do not participate in workplace learning (WPL).

### Data collection

The clicker questionnaire was pilot-tested with six radiographers and four students from a radiology department not participating in the main study to address any potential problems arising from the clicker system and the questionnaire. During the pilot test, the participants could not select more than one answer for questions indicated as such. Thus, the necessary changes to the questionnaire loaded on the clicker system were made before the commencement of data collection for the main study.

The clicker questionnaire was in English because the radiographers received their training in English at a university in South Africa. The clicker system uses a unique code for each participant to protect their identity, ensuring anonymous participation.

Data collection sessions were arranged with different departments. For some 2nd-year students, data were collected at the academic institution as they had classes during the data collection period, and the data collection did not interrupt their studies. A computer with a receiver was used to capture the information from the clicker devices. All the clickers were set to a universal operation setting channel, allowing the receiver to transmit the participants’ answers to the TurningPoint programme. Data were collected from 20 to 27 October 2020, when South Africa was at lockdown level 1, at the participating hospitals during the COVID-19 pandemic. The clicker questionnaire was administered in the presence of the researchers to ensure that the participants did not discuss their answers with one another.

### Data analysis

After collecting the data, the results were saved in Microsoft Excel format using the TurningPoint programme. In total, seven sessions of data collection were conducted. The software analysed the quantitative data, which presented the results as descriptive statistics. The results of the different sessions were sent to a statistician for data analysis to ensure validity using SAS version 9.4 (SAS Institute Inc., Cary, NC, United States). The statistician calculated descriptive statistics, including frequencies and percentages, for the categorical data. The Wilcoxon two-sample test was used to calculate the significance of differences between the median values regarding the participants’ knowledge control. The Kruskal-Wallis test was performed to calculate the significance of differences between the experiences, training cycles of infection knowledge of the participants, respectively. According to the Shapiro-Wilk test for normality, the data did not follow a normal distribution. Consequently, non-parametric tests were used.

### Validity and reliability

The validity and reliability of the study were ensured through pilot-testing of the clicker questionnaire before the main study. Content validity was assessed during the pilot test to determine whether the questions appropriately measured radiographers’ knowledge of infection control. The pilot test was used as a tool to determine if the questionnaire covered the content related to infection control. Furthermore, the questionnaire’s content was based on literature, which contributed to content validity. In this study, Cronbach’s alpha (α) was used to calculate the internal consistency of the 20 items testing the level of knowledge in the questionnaire. George and Mallery (2003:231) provide the following rules of thumb for the interpretation of α: ‘_ > 0.9 – Excellent, _ > 0.8 – Good, _ > 0.7 – Acceptable, _ > 0.6 – Questionable, _ > 0.5 – Poor, and _ < 0.5 – Unacceptable’. A reliability analysis on the level of knowledge comprising 20 items resulted in a Cronbach’s alpha (α) of 0.44. The scale had poor internal consistency (α < 0.05). As a result of the poor internal consistency, the 20 questions were grouped into two subscales, namely infection cycle (12 questions: questions 1–6, 10, 12, 17–20) and breaking the infection cycle (eight questions: questions 7–9, 11, 13–16). The Cronbach’s alpha (α) was 0.41 and 0.26 for the subscales infection cycle and breaking the infection cycle, respectively, which is also an indication of poor internal consistency.

### Ethical considerations

Ethics approval to conduct the study was obtained from the University of the Free State Health Sciences Research Ethics Committee (HSREC) (reference number UFS-HSD2020/1407/ 2710) and the Free State province Department of Health. Permission was also obtained from the heads of the radiology departments at the participating hospitals as well as from the senior director of Institutional Planning and Quality Enhancement at the institution where the students were enrolled. The radiographers and diagnostic radiography students received an information document on the study. The participants then completed and signed an informed consent document.

## Results and discussion

### Characteristics

A total of 106 participants fully completed the questionnaire with clickers. The demographic characteristics of the participants are presented in Table 1, showing that most participants were student radiographers in diagnostic radiography (*n* = 75; 70.8%). Two participants did not specify their position and qualifications. This could be because of technicalities they might have experienced during completion of the questionnaire, which was not communicated to the researchers. As a result, the data of these two participants were not included in the study. The 31 (29.2%) participants who were not students were asked to select their current position in the radiology department from a list of options. The majority indicated that they were qualified diagnostic radiographers (*n* = 27; 25.5%). Only four (3.8%) radiographers had a master’s degree, whereas 21 (19.8%) had a diploma in diagnostic radiography. Most qualified participants indicated 6 year–10 years of professional experience (*n* = 9/31; 29.0%). The majority of the participants were between 20 and 30 years of age (*n* = 82/105; 78.1%). A noteworthy finding was that one of the 31 qualified participants selected the option ‘not applicable’ in relation to the years of professional experience. A possible reason for this selection could be that the individual did not consider the community service year as work experience but as part of obtaining a qualification which is required for registration with the HPCSA.

**TABLE 1:** Characteristics of the participating diagnostic and student radiographers (*N* = 106).

Variable	*n*	%
**Current position in the radiology department**		
Radiographers	31	29.2
Qualified radiographer	27	25.5
Supplementary radiographer	3	2.8
Community service radiographer	1	0.9
Student radiographers	75	70.8
2nd-year radiography student	28	26.4
3rd-year radiography student	24	22.6
4th-year radiography student	23	21.7
Not specified	2	1.9
**Highest qualification**		
Diploma	21	19.8
Bachelor’s degree	11	10.4
Master’s degree	4	3.8
Student radiographer	68	64.2
Not specified	2	1.9
**Professional experience of radiographers (years) (*n* = 31)**		
1–5	5	16.1
6–10	9	29.0
11–20	5	16.1
21–30	6	19.4
≥ 31	5	16.1
Not applicable	1	3.2
**Age (years) (*n* = 105)**		
20–30	82	78.1
31–40	8	7.6
41–50	8	7.6
≥ 50	7	6.7

### Knowledge of infection control

**FIGURE 1:**
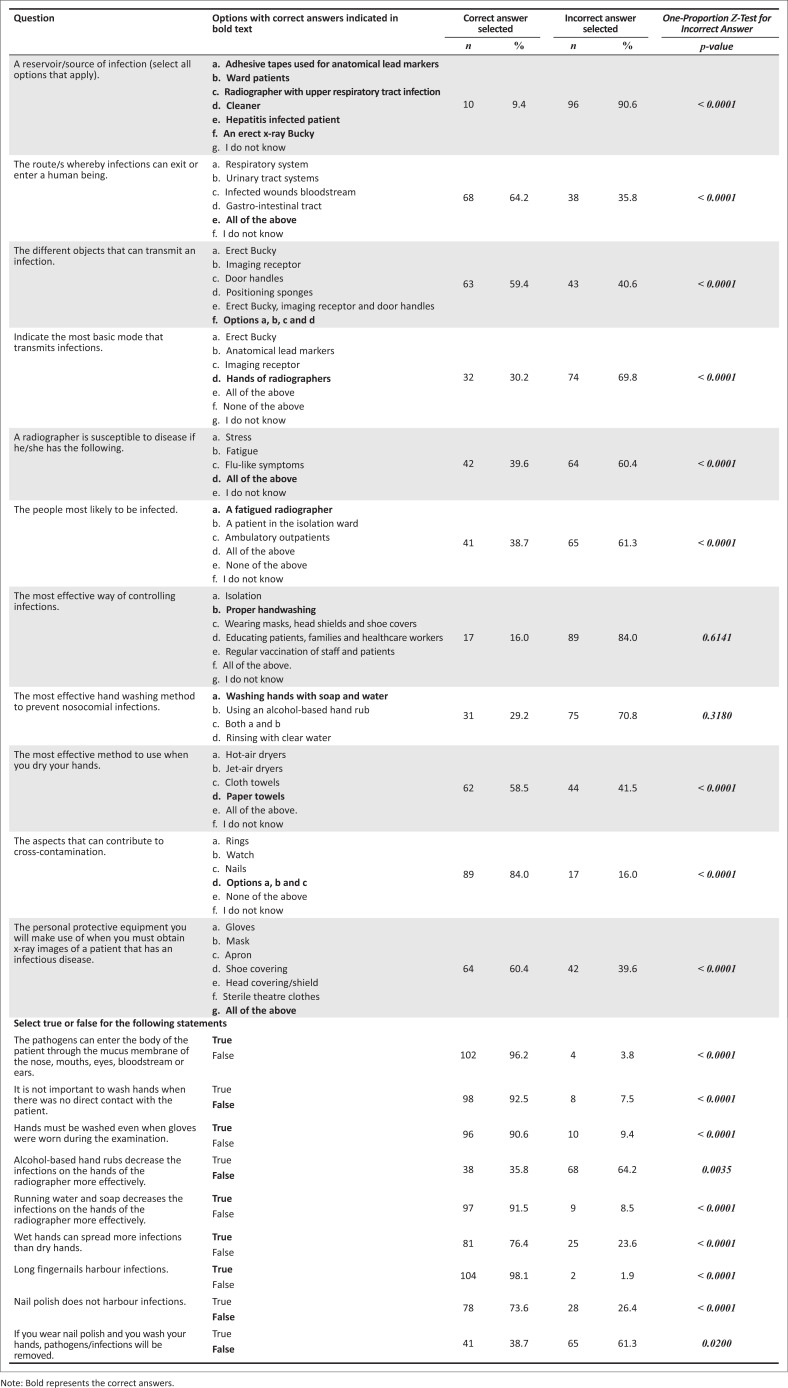
Knowledge questions with possible answers and the number of participants who correctly answered each question (*N* = 106).

From Figure 1, it is evident that several participants had little knowledge of aspects such as the source of infection (*n* = 10; 9.4%), the basic mode to transmit infection (*n* = 32; 30.2%), the most effective way of controlling infections (*n* = 17; 16.0%) and the most effective handwashing method (*n* = 31; 29.2%). During a pandemic, one would have expected that the participants would answer the above-mentioned questions correctly, seeing that so much information was readily available on infection cycle and ways to control infections. Sixty-eight (64.2%) participants were aware of the different routes of infections, while 81 (76.4%) participants knew that wet hands spread infections more readily than dry hands. Ninety-seven (91.5%) participants agreed that washing your hands with water and soap decreases infections more effectively, whereas 104 (98.1%) participants were aware that long fingernails harbour infections.

Based on the results summarised in Figure 1, the participants performed poorly on specific questions, which could result in suboptimal practices in the radiology departments. Healthcare professionals’ hands are the most basic pathway of transmitting infections. Hence, proper hand hygiene is the most effective way to break the cycle of infection transmission (Ahmed et al. 2017; Gammon & Hunt 2019; WHO 2021). However, only 16.0% of the participants were aware of this basic practice.

Less than 30% of the participants knew that washing your hands with water and soap is the most effective handwashing method, as shown in Figure 1, because it removes spores and microorganisms (Ehrlich & Coakes 2017). In contrast, alcohol hand rub removes only certain bacteria and viruses, not bacterial and fungal spores or alcohol-resistant viruses (Prajapati, Desai & Chandarana 2022). The question could have misled the participants as it did not specify the most effective handwashing method for either soiled or unsoiled hands. Both the WHO (2021) and the CDC (2024) recommend washing hands with soap and water when they are visibly soiled with dirt or bodily fluids, while alcohol-based hand rub should only be used when hands are not visibly dirty. It is possible that the participants achieved a poor score for that specific question because they might have considered both handwashing methods (alcohol-based hand rub and water and soap) to be effective. Wet hands or not adequately dried hands spread infections more readily than dry hands, which 81 (76.4%) participants answered correctly. The most effective method to dry your hands is using paper towels, which effectively remove bacteria from the hands (Gammon & Hunt 2019; Suen et al. 2019), a knowledge that was known by less than 60% of the participants.

As shown in Figure 1, most participants were aware that long nails harbour infectious agents (CDC 2024). However, despite more than 70% of the participants knowing that nail polish could harbour microorganisms, less than 40% indicated that infectious material is not removed from nail polish even if you have washed your hands. Cimon and Featherstone (2017) reported that no substantial evidence was available from previous research investigating the transmission of infections from nail polish. Therefore, it is reasonable to conclude that the participants in this study also felt that wearing nail polish does not pose a risk of infections when hands are washed properly. However, wearing no nail polish is considered a safety measure to prevent the risk of infection transmitted by healthcare workers (Suen et al. 2019).

The human body is considered a major reservoir of infectious microorganisms as this particular environment provides three essential elements for microbes to flourish: moisture, nutrients and a suitable temperature (Ehrlich & Coakes 2017). However, adhesive tapes are also considered reservoirs of infection (Ilyas et al. 2019). The erect X-ray Bucky, which forms part of healthcare equipment, is also considered a source of infections, as highlighted by Picton-Barnes, Pillay and Lyall (2020). The participants might have reasoned that only the human body could be considered a reservoir of infections, which might explain why less than 10% provided the correct answer.

### Impact of training on knowledge

At the time of the study, only 58.5% of the participants had received infection control training within the past 6 months, a proportion that is higher than previously reported in similar studies (Abdelrahman et al. 2017) or lower than the findings of another study (Ahmed et al. 2017). The majority of participants (*n* = 64; 60.4%) in this study received training within 1 to 6 months before the study. Only 25.5% of the participants received training in the preceding month (Table 2). Considering that the study was conducted during the COVID-19 pandemic, it would be expected that infection control training was provided more consistently. The apparent lack of such training is therefore concerning. It was noteworthy that training did not impact knowledge because participants who did not receive training had the same median score as those who did receive training on infection control (Table 3). The highest score of 80% was achieved by a participant who had not received training. These findings were contrary to those of other studies which found that individuals who received training had better knowledge of infection control concepts than those who had not been trained (Abdelrahman et al. 2017; Ahmed et al. 2017).

**TABLE 2:** Training and self-rated level of knowledge among the participants regarding infection control (*N* = 106).

Variable	*n*	%	Median	Range
**Received any recent training in infection control**				
Yes	62	58.5	60	50–65
No	44	41.5	60	50–65
**Period since last infection control training**				
1 month ago	27	25.5	-	-
Between 3 and 6 months ago	37	34.9	-	-
Between 6 months and a year ago	16	15.1	-	-
Between 1 year and 5 years ago	9	8.5	-	-
Longer than 5 years ago	5	4.7	-	-
I never received training in infection control	12	11.3	-	-
**Self-rated level of knowledge on the cycle of infection**				
Low	0	0	-	-
Adequate	66	62.3	60	50–65
Excellent	40	37.7	60	53–65

**TABLE 3:** Comparisons of scores regarding infection control knowledge per variable (*N* = 106).

Variable	Score (%)			*p*-value	CI %
	Median	Range	IQR		
Overall knowledge scores (*n* = 106)	60	25–80	50; 65	-	-
**Position**	-	-	-	0.8548	-
Radiographers (*n* = 31)	60	40–70	50; 65	-	-
Student radiographers (*n* = 75)	60	25–80	50; 65	-	-
**Years experience**	-	-	-	0.3647	-
1–5 years (*n* = 5)	60	50–65	55; 60	-	51–65
6–10 years (*n* = 9)	60	40–70	50; 65	-	51–67
11–20 years (*n* = 5)	50	40–60	45; 60	-	40–62
21–30 years (*n* = 6)	57.5	45–70	45; 65	-	46–68
≥ 31 years (*n* = 5)	65	50–70	60; 65	-	53–71
**Level of qualification**	-	-	-	-	-
Radiographers	-	-	-	0.6944	-
Qualified (*n* = 27)	60	40–70	50; 65	-	-
Supplementary (*n* = 3)	60	40–60	40; 60	-	-
Community service (*n* = 1)	60	60–60	60; 60	-	-
Student radiographers	-	-	-	0.9262	-
2nd-year radiography (*n* = 28)	60	35–80	50; 65	-	-
3rd-year radiography (*n* = 24)	60	25–75	50; 65	-	-
4th-year radiography (*n* = 23)	60	30–70	50; 65	-	-
**Previous training received**	-	-	-	0.9249	-
Yes (*n* = 62)	60	35–75	50; 65	-	-
No (*n* = 44)	60	25–80	50; 65	-	-
**Self-rated level of knowledge**	-	-	-	0.5174	-
Adequate (*n* = 66)	60	35–80	50; 65	-	-
Excellent (*n* = 40)	60	25–75	52.5; 65	-	-

CI, confidence interval; IQR, interquartile range.

### Knowledge of infection control based on professional position

The qualified radiographers scored higher than the community service and supplementary radiographers, although this difference was not statistically significant (*p* = 0.6944). No significant differences were noted between the knowledge of qualified radiographers and that of students (*p* = 0.8548). As shown in Table 3, the 2nd-year students performed slightly better than the other year groups in terms of the maximum score obtained, although the difference between the years of study was not statistically significant (*p* = 0.9262).

The 2nd-year students had the highest maximum score (80%) compared to the other year groups. During the 1st year of study, diagnostic radiography students receive theoretical classes on infection control, which may explain why the 2nd-year students performed better than those in other year groups.

Furthermore, there were no significant differences between participants who had received training and those who had not, suggesting that the current training may not be sufficiently effective in improving knowledge. It was noteworthy that among participants with no previous training, the highest score obtained was 80%, compared to 75% in the group that received training.

The median score for both qualified radiographers and student radiographers was 60%, which was lower than previously reported (Abdelrahman et al. 2017; Nyirenda et al. 2018).

### Impact of experience on knowledge

The lowest median scores (50%, range 40% – 60%; 57.5%, range 45% – 70%) were observed in participants with 11–20 and 21–30 years’ experience in diagnostic radiography, respectively. No statistically significant association was observed between years of experience and knowledge scores (*p* = 0.3647), as shown in Table 3, which is similar to previous findings (Nyirenda et al. 2018). However, subgroup medians varied: radiographers with ≥ 31 years of experience had higher median scores (65%) compared with mid-career groups (50% – 57.5%). Confidence intervals around these estimates were wide, reflecting the small subgroup sizes (e.g. *n* = 5 for ≥ 31 years). These results should, therefore, be interpreted with caution.

The knowledge among the subgroups with different years of experience was compared with each other using the Wilcoxon two-sample test. The p-value between radiographers with 1–5 years of experience and those with 6–10 years was 0.68. The same trend was observed among the other subgroups with varying years of experience, with no significant difference noted. Statistically, the years of experience did not influence the knowledge of the participants.

While our analysis did not identify a statistically significant association between years of experience and knowledge, this should not be interpreted as definitive evidence of no effect. The small subgroup sizes, particularly among radiographers with ≥ 31 years of experience, limited statistical power and increased the risk of a Type II error (failing to detect an effect that may exist). Moreover, this study was based on a convenience sample, which restricts the generalisability of the findings to the broader radiographer population. Taken together, the results should be considered exploratory and hypothesis-generating. The observed higher median scores among the most experienced subgroup may warrant further investigation in larger, randomly sampled studies.

### Self-rated level of knowledge compared with actual knowledge scores

All participants rated their knowledge of infection control as either adequate or excellent. During the COVID-19 pandemic, one would expect radiographers to have knowledge of infection control of 75% or above. However, they achieved a median score of 60% on the vast majority of the 20 questions addressing infection control (Table 3). Most participants indicated that their level of knowledge was adequate, with 40 (37.7%) regarding their knowledge of infection control as excellent (Table 2). However, only one participant (0.9%) achieved a score of 80% on the knowledge-based questions, compared to the median score of 60%. The lowest score was 35% for those who regarded themselves as having adequate knowledge and 25% for those with perceived excellent knowledge. Those participants who indicated that their knowledge was excellent scored between 52.5% and 65%, and only one participant scored 75%. What is perceived as adequate or excellent might not necessarily reflect the true level of knowledge. Moreover, based on the overall scores of the participants, improvement is required for all the radiographers who participated in the study.

### Recommendations

Regular infection control in-service training, which involves both theoretical and practical sessions (Abdelrahman et al. 2017), must be implemented to raise awareness among radiographers about the risks of infection. Preventive measures to reduce the risk of infection transmission should be complemented with regular infection control audits throughout the year, as implemented in China at the onset of the COVID-19 pandemic (Chen et al. 2020). Specifically, based on the results of this study, the infection control audits should focus on knowledge. This approach would be beneficial for radiographers to determine adherence to or compliance with infection control guidelines required to reduce the risk of infections and cross-contamination.

### Limitations

A major limitation of the study is that most of the participants were student radiographers; thus, qualified radiographers were under-represented. As a result, there might be bias in the results presented. A limitation of the study was that the questions related to handwashing methods did not specify which method was effective for soiled and unsoiled hands. Consequently, participants could have been confused when answering questions about effective handwashing methods. Another limitation was that the study was only conducted at three public sector radiology departments and did not include private sector practices. The sample size was limited because some radiographers were on annual leave, worked night shifts, or refused to participate. Additionally, the data of two participants were excluded because of incomplete information. Social desirability was also noted in the study. The participants rated their self-knowledge very highly compared to what their knowledge actually was. Subsequently, this led to participant bias within the study. Furthermore, only infection control knowledge was assessed, and the practices thereof were not assessed in this study.

The confidence intervals were also calculated. However, the validity of confidence intervals depends heavily on how the sample is collected (Hedt & Pagano 2011). For this study, convenience sampling was also used, which does not involve random sample selection. This lack of randomness means the sample may systematically introduce bias. The subgroups in this study were small. With small sample sizes, confidence intervals become wide and imprecise, reducing the certainty of effect estimates and undermining confidence in scientific conclusions, which was a limitation in this study (Corty & Corty 2011).

## Conclusion

The results of the study showed that the participants had low to adequate knowledge of infection control during the pandemic, which could negatively impact infection control behaviour. The duration of experience and receiving training may not have impacted the participants’ knowledge. Therefore, further research on infection control practices is recommended. Radiology departments should consider having regular infection control audits to promote compliance among radiographers.
